# Evaluation of Infrazygomatic Crest Bone Thickness for Extra-Alveolar Temporary Anchorage Device Placement: A Retrospective Cone-Beam Computed Tomography Study

**DOI:** 10.7759/cureus.114627

**Published:** 2026-08-16

**Authors:** Zaheer Basha, Imtiyaz Azam Hebbal, Rafi Shaik, Shaik Sameeulla, Vamsi Krishna Reddy, Hanish Shashidhar

**Affiliations:** 1 Department of Orthodontics, Ozone Dental Clinic, Al Khobar, SAU; 2 Department of Orthodontics, Dar As Sihha Medical Center, Dammam, SAU; 3 Department of Public Health Dentistry, Narayana Dental College and Hospital, Nellor, IND; 4 Department of Oral Medicine and Radiology, Chadalawada Krishna Srinivasa (CKS) Teja Institute of Dental Sciences and Research, Tirupati, IND; 5 Department of Prosthodontics, Narayana Dental College and Hospital, Nellore, IND; 6 Department of General Medicine and Critical Care, Manorama Super Specialty Hospital, Nizamabad, IND

**Keywords:** cone-beam computed tomography, infrazygomatic crest, malocclusion, maxilla, temporary anchorage devices

## Abstract

Introduction: The infrazygomatic crest (IZC) is a favorable site for extra-alveolar temporary anchorage device (TAD) placement during orthodontic treatment. However, anatomical variations in bone thickness necessitate individualized preoperative assessments to ensure safe and predictable TAD placement. This retrospective cone-beam computed tomography (CBCT) study evaluated IZC bone thickness at standardized levels and compared measurements according to sex and skeletal malocclusion.

Methods: A total of 150 pre-treatment CBCT scans (300 IZC sites) of patients aged 18-35 years were analyzed. Total bone thickness, cortical bone thickness, and available bone thickness were measured at 11 mm and 13 mm apical to the cementoenamel junction of the maxillary first molar along a 70^o^ insertion trajectory. Measurements were compared between sexes using independent t-tests and among skeletal malocclusion groups (Class I, II, and III) using one-way analysis of variance (ANOVA) with Tukey's post hoc test.

Results: Measurements at 13 mm showed greater total (7.85 mm) and available bone thickness (6.37 mm) than those at 11 mm (6.42 mm and 5.14 mm, respectively). Males demonstrated significantly greater bone thickness than females at both levels (p<0.001). Skeletal Class III subjects exhibited the greatest total and available bone thickness, followed by Class I and Class II subjects (p<0.05), whereas cortical bone thickness did not differ significantly among the groups.

Conclusion: The IZC provided adequate bone for extra-alveolar TAD placement, with a 13 mm level offering greater bone availability. Significant variations according to sex and skeletal malocclusion supported individualized CBCT-based treatment planning to optimize the safety and predictability of TAD placement.

## Introduction

Temporary anchorage devices (TADs) have transformed contemporary orthodontic treatment by providing reliable skeletal anchorage with minimal dependence on patient compliance [1]. Among the various extra-alveolar insertion sites, the infrazygomatic crest (IZC) has emerged as one of the most favorable locations for maxillary anchorage because it allows placement away from the dental roots while providing stable support for complex tooth movements such as en-masse retraction, distalization, intrusion, and correction of sagittal discrepancies [2]. The successful placement and long-term stability of IZC screws are primarily influenced by the quantity and quality of available bone, particularly cortical bone thickness [3].

The anatomical characteristics of the IZC exhibit considerable individual variation depending on sex, skeletal morphology, facial patterns, and craniofacial anatomy. Inadequate bone thickness may compromise primary stability and increase the risk of screw failure or inadvertent injury to adjacent anatomical structures, including the maxillary sinus and tooth roots [3,4]. Therefore, an accurate preoperative assessment of IZC bone morphology is essential for selecting the optimal insertion site and angulation.

Cone-beam computed tomography (CBCT) has been widely established as an effective modality for evaluating localized bone thickness variations across different skeletal malocclusion patterns [5]. CBCT has become the imaging modality of choice for evaluating craniofacial hard tissues because it provides high-resolution three-dimensional images with relatively low radiation exposure compared with conventional computed tomography [4,6]. CBCT enables precise measurement of total bone thickness, cortical bone thickness, and available bone at different levels apical to the cementoenamel junction, thereby facilitating individualized treatment planning for extra-alveolar TAD placement.

Although previous investigations have reported IZC bone dimensions in different populations, variations among ethnic groups and skeletal malocclusion patterns limit the generalizability of these findings [4,6]. Furthermore, information regarding differences according to sex and skeletal classification remains inconsistent. Hence, population-specific CBCT data are required to establish clinically relevant guidelines for a safe and predictable IZC screw placement. Furthermore, evaluating three-dimensional maxillary and sinus anatomical variations across different sagittal skeletal discrepancies is essential for predicting bone availability, as highlighted by Azizia et al. [7]. Therefore, the present retrospective CBCT study was undertaken to evaluate IZC bone thickness at standardized levels adjacent to the maxillary first molar and to determine whether these measurements differ according to sex and skeletal malocclusion, thereby assisting clinicians in selecting the most appropriate site for extra-alveolar anchorage.

The primary objective was to measure the total bone thickness, cortical bone thickness, and available bone thickness at 11 mm and 13 mm apical to the cementoenamel junction of the maxillary first molar at a 70° insertion trajectory. The secondary objective was to compare these measurements according to sex and skeletal malocclusion (Class I, Class II, and Class III) in order to identify anatomical variations that may influence the optimal site for safe and predictable IZC TAD placement.

## Materials and methods

This retrospective observational study was conducted in the Department of Orthodontics and Dentofacial Orthopedics, Narayana Dental College and Hospital, Nellore, Andhra Pradesh, India, after obtaining approval from the Institutional Ethics Committee (NDCPG-2021-22/IEC/2021 dated December 27, 2021). The study was carried out between January 2022 and June 2022 using archived CBCT records acquired during the preceding three years (January 2019 to December 2021) from the departmental database. Two investigators (IAH and RS) screened all available CBCT records for eligibility and retrieved scans that fulfilled the predefined inclusion criteria. This study was conducted in accordance with the ethical principles outlined in the Declaration of Helsinki.

The sample size was estimated using the G*Power software (version 3.1.9.7; Heinrich Heine University, Düsseldorf, Germany) based on the F-test family (one-way analysis of variance (ANOVA), fixed effects, omnibus). Considering a medium effect size (Cohen's f=0.25), significance level (α) of 0.05, and statistical power of 80%, the minimum required sample size was calculated to be 135 CBCT scans [3]. To compensate for possible exclusions owing to poor image quality, motion artifacts, or incomplete records, the final target sample size was increased to 150 CBCT scans, which was considered adequate to achieve the desired statistical power.

The inclusion criteria were CBCT scans of subjects aged 18-35 years with fully erupted permanent dentition, fully erupted maxillary first molars, clearly identifiable IZC anatomy bilaterally, and high-quality CBCT images free from motion or metallic artifacts. CBCT scans of subjects with a history of orthodontic treatment involving skeletal anchorage, craniofacial syndromes or developmental anomalies, cleft lip and palate, maxillofacial trauma, orthognathic surgery, pathological lesions involving the maxilla, severe periodontal bone loss affecting the maxillary first molar region, missing maxillary first molars, or poor-quality CBCT images were excluded from the study.

All CBCT scans were acquired using the CS 9600 Cone Beam 3D Imaging System (Carestream Dental LLC, Atlanta, Georgia, USA) following a standardized imaging protocol. The exposure parameters included 90 kVp, 4-10 mA, a voxel size of 0.08 mm, and a field of view of 5 x 5 cm. Digital Imaging and Communications in Medicine (DICOM) datasets were imported into CS Imaging Software (Version 8.0, Carestream Dental LLC, Atlanta, Georgia, USA) for image reconstruction, orientation, and linear measurements. Before analysis, all scans were reoriented by aligning the Frankfort horizontal plane parallel to the floor and the midsagittal plane perpendicular to the horizontal plane to ensure uniformity of the measurements.

**Figure 1:**
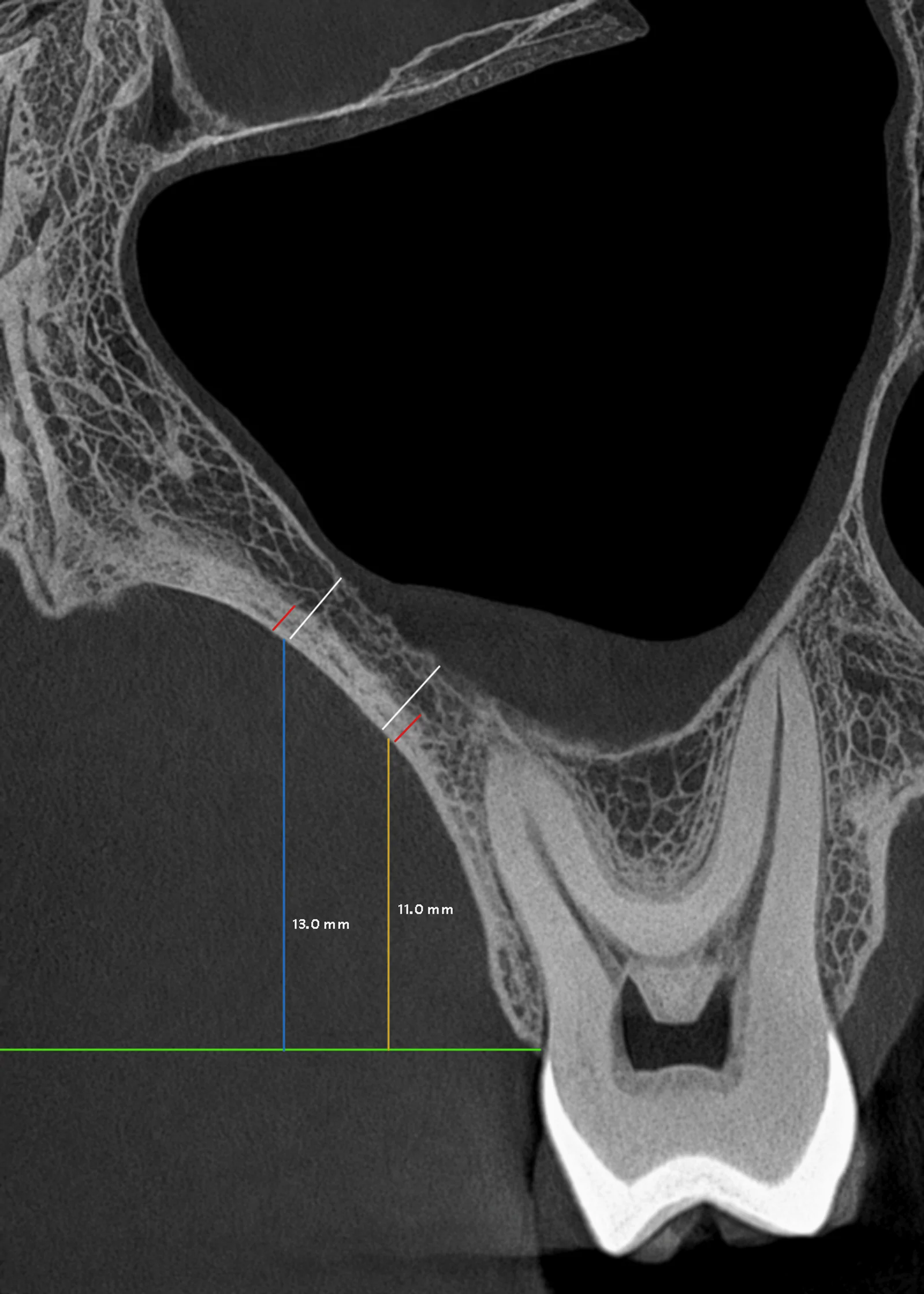
Measurement of infrazygomatic bone thickness at 11 mm (yellow line) and 13 mm distance (blue line) Green line: Standard stable line from cementoenamel junction of maxillary first molar tooth; white line: total bone thickness; red line: cortical bone thickness Original image of the study sample CBCT images were acquired using a limited field of view (5 × 5 cm) at 90 kVp, 7 mA, with an isotropic voxel size of 0.08 mm. Images were reconstructed using a high-resolution bone algorithm with a slice thickness of 0.2 mm and exported in DICOM format for analysis. CBCT, cone-beam computed tomography; DICOM, Digital Imaging and Communications in Medicine.

IZC measurements were performed on standardized coronal CBCT sections passing through the maxillary first molar. Measurements were obtained bilaterally at 11 mm and 13 mm apical to the cementoenamel junction of the maxillary first molar along a 70^o^ insertion trajectory relative to the occlusal plane, simulating the recommended clinical angulation for placement of extra-alveolar TADs [3,4]. At each level, total bone thickness was measured as the distance from the outer buccal cortical surface to the inner cortical boundary adjacent to the maxillary sinus. Cortical bone thickness represents the thickness of the buccal cortical plate, and available bone thickness represents the cancellous bone available for implant engagement after excluding the cortical component (Figure 1). Measurements from both the right and left sides were recorded for each participant, and the average values were used for subsequent statistical analyses.

CBCT evaluations were performed independently by three authors (ZB, SS, and HS) at three separate workstations. Each examiner was blinded to the patients' demographic details, skeletal classifications, and measurements recorded by the other observers throughout the study period. Before the commencement of the study, all examiners underwent calibration using a set of representative CBCT scans to standardize the measurement protocol. To determine intra-observer reliability, 20% of the randomly selected CBCT scans were re-evaluated by each examiner after a two-week interval without access to the initial measurements. Inter-observer reliability was assessed by comparing measurements obtained independently by the three observers. The intraclass correlation coefficient (ICC) demonstrated excellent reproducibility, with intra-observer ICC values ranging from 0.88 to 0.94 and inter-observer ICC values ranging from 0.87 to 0.92, indicating excellent agreement and reliability of the measurement protocol.

Statistical analysis was performed independently by another author (VKR), who remained blinded to the identity of the participants and the CBCT measurements recorded by the examiners. Data analysis was performed using IBM SPSS Statistics for Windows, version 25.0 (IBM Corp., Armonk, New York, USA). The normality of the data distribution was assessed using the Shapiro-Wilk test. Continuous variables are expressed as mean ± standard deviation, whereas categorical variables are presented as frequencies and percentages. The IZC area was measured bilaterally on 150 CBCT scans. As side-to-side differences were not statistically significant, the right and left measurements were pooled and averaged for each scan, resulting in 150 independent values for statistical evaluation. Differences between male and female subjects were evaluated using an independent-samples t-test. Comparisons among skeletal Class I, Class II, and Class III malocclusion groups were performed using one-way ANOVA, followed by Tukey's honestly significant difference (HSD) post hoc test for pairwise comparisons. A two-tailed p<0.05 was considered statistically significant for all analyses.

## Results

In total, 150 CBCT scans were included in the final analysis, providing 300 IZC sites for evaluation. The study population had a mean age of 24.6 ± 4.8 years (range: 18-35 years) and comprised 72 males (48.0%) and 78 females (52.0%). Based on skeletal malocclusion, 68 (45.3%) patients were classified as Class I, 54 (36.0%) as Class II, and 28 (18.7%) as Class III. Demographic and clinical characteristics of the study participants are shown in Table 1.

**Table 1:** Demographic and clinical characteristics of the study participants Values are presented as mean ± standard deviation (SD) for continuous variables and number (percentage) for categorical variables. N, participants.

Variable		Value
Total scans evaluated, N (%)	Right side	150 (100.0)
	Left side	150 (100.0)
Age, years	Mean ± SD (range)	24.6 ± 4.8 (18-35)
Sex, N (%)	Male	72 (48.0)
	Female	78 (52.0)
Skeletal, N (%)	Class I	68 (45.3)
	Class II	54 (36.0)
	Class III	28 (18.7)

**Figure 2:**
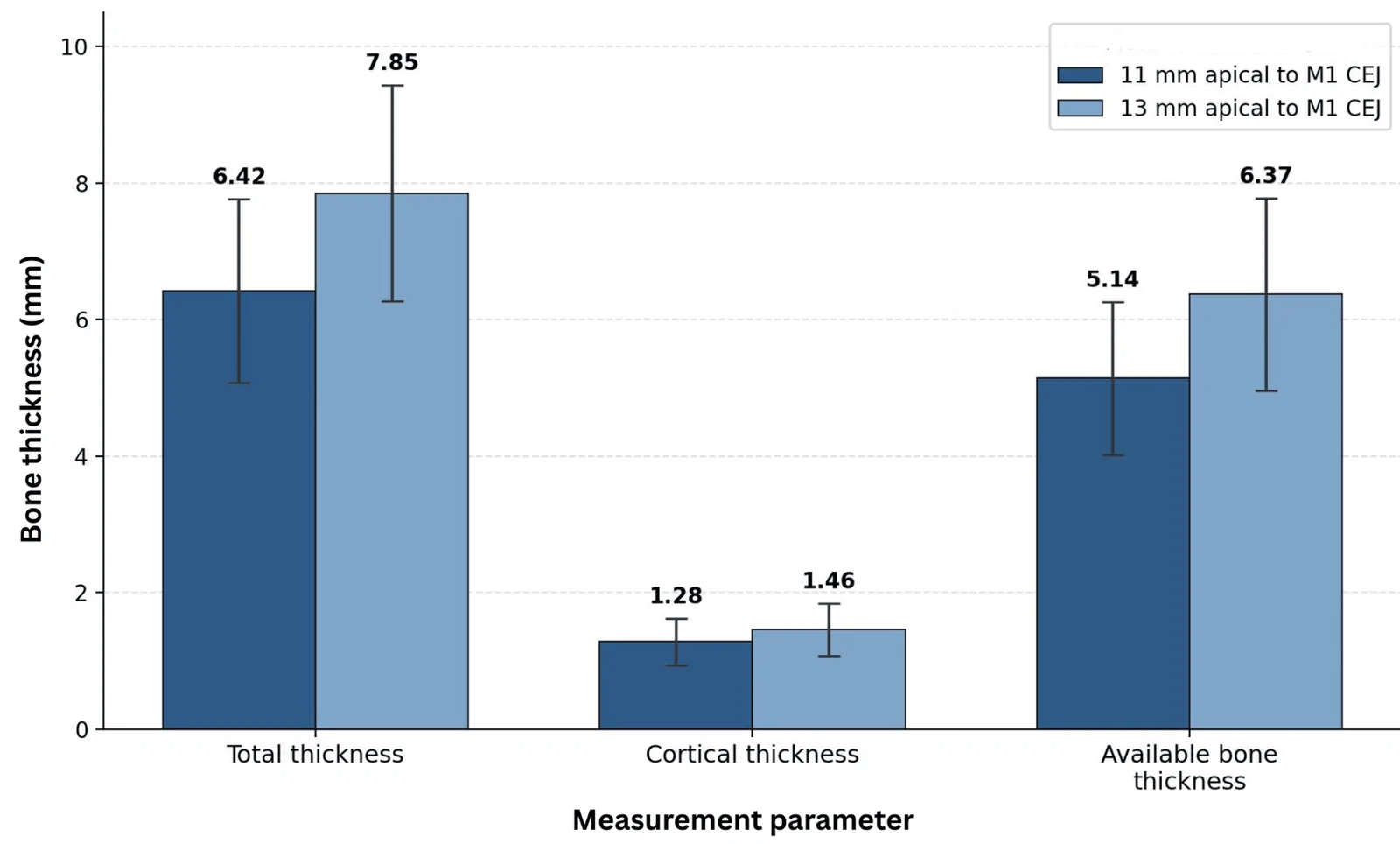
Mean outcome parameters (bone thickness in mm) at 11 and 13 mm apical to cementoenamel junction The infrazygomatic area was measured bilaterally on 150 CBCT scans. As side-to-side differences were not statistically significant, the right and left measurements were pooled and averaged for each scan, resulting in 150 independent values for statistical evaluation. CBCT, cone-beam computed tomography.

Figure 2 illustrates the overall IZC bone thickness measurements obtained from the study sample at 11 mm and 13 mm apical to the cementoenamel junction of the maxillary first molar. At the 11 mm level, the mean total bone thickness was 6.42 mm, the cortical bone thickness was 1.28 mm, and the available bone thickness was 5.14 mm. At the 13 mm level, the corresponding values increased to 7.85 mm, 1.46 mm, and 6.37 mm, respectively. Overall, measurements obtained at 13 mm apical to the cementoenamel junction demonstrated greater total bone thickness, cortical bone thickness, and available bone thickness than those recorded at 11 mm, indicating that the more apical region of the IZC provides a greater amount of bone for extra-alveolar TAD placement. Error bars represent standard deviations, demonstrating the variability of the measurements within the study population.

A comparison of IZC bone thickness between male and female subjects is summarized in Table 2. Male participants demonstrated significantly greater total, cortical, and available bone thicknesses than female participants at both measurement levels. The differences in total bone thickness and available bone thickness were highly significant (p<0.001), whereas cortical bone thickness also showed statistically significant differences (p<0.05), indicating greater bone availability in males for IZC screw placement.

**Table 2:** Comparison of infrazygomatic crest bone thickness according to sex Values are presented as mean ± SD. Comparisons between male and female subjects were performed using the independent-samples t-test. *p<0.05 was considered statistically significant. CEJ, cementoenamel junction; M1, maxillary first molar; SD, standard deviation.

Parameter	Height	Male (mean ± SD, mm)	Female (mean ± SD, mm)	t statistics	p-value	Effect size (Cohen’s d)
Total thickness	11 mm apical to M1 CEJ	6.78 ± 1.29	6.09 ± 1.33	3.92	<0.001*	0.79
	13 mm apical to M1 CEJ	8.24 ± 1.49	7.49 ± 1.58	3.65	<0.001*	0.74
Cortical thickness	11 mm apical to M1 CEJ	1.34 ± 0.33	1.23 ± 0.34	2.44	0.015*	0.49
	13 mm apical to M1 CEJ	1.52 ± 0.37	1.41 ± 0.38	2.20	0.028*	0.44
Available bone thickness	11 mm apical to M1 CEJ	5.46 ± 1.09	4.85 ± 1.08	4.28	<0.001*	0.87
	13 mm apical to M1 CEJ	6.74 ± 1.36	6.03 ± 1.39	3.94	<0.001*	0.80

A comparison of IZC bone thickness among skeletal Class I, Class II, and Class III malocclusions is presented in Table 3. Significant differences were observed in the total bone thickness and available bone thickness at both the 11 mm and 13 mm levels (p<0.05). In contrast, cortical bone thickness did not differ significantly among the skeletal groups. Overall, skeletal Class III subjects exhibited the greatest total and available bone thickness, followed by Class I and Class II subjects, respectively.

**Table 3:** Comparison of infrazygomatic crest bone thickness among skeletal malocclusion groups Values are presented as mean ± standard deviation. Comparisons among skeletal Class I, Class II, and Class III groups were performed using one-way analysis of variance followed by Tukey's honestly significant difference post hoc test. *p<0.05 was considered statistically significant. CEJ, cementoenamel junction; M1, maxillary first molar.

Parameter	Height	Class I (n=68)	Class II (n=54)	Class III (n=28)	F-value	p-value	Effect size (η^2^)
Total thickness	11 mm apical to M1 CEJ	6.51 ± 1.28	6.05 ± 1.31	6.98 ± 1.40	4.61	0.011*	0.059
	13 mm apical to M1 CEJ	7.92 ± 1.50	7.38 ± 1.55	8.44 ± 1.62	4.15	0.017*	0.053
Cortical thickness	11 mm apical to M1 CEJ	1.30 ± 0.33	1.20 ± 0.32	1.36 ± 0.36	2.58	0.078	0.034
	13 mm apical to M1 CEJ	1.48 ± 0.37	1.38 ± 0.36	1.55 ± 0.40	2.31	0.101	0.030
Available bone thickness	11 mm apical to M1 CEJ	5.21 ± 1.10	4.85 ± 1.15	5.62 ± 1.18	3.94	0.021*	0.051
	13 mm apical to M1 CEJ	6.45 ± 1.38	6.02 ± 1.42	6.89 ± 1.44	3.72	0.026*	0.048

Pairwise comparisons using Tukey's HSD post hoc test are presented in Table 4. Significant differences were primarily observed between Class II and Class III subjects and between Class I and Class III subjects for the total bone thickness and available bone thickness, whereas no significant pairwise differences were identified for the cortical bone thickness at either measurement level.

**Table 4:** Pairwise comparisons of infrazygomatic crest bone thickness among skeletal malocclusion groups using Tukey's post hoc test Values represent pairwise comparisons obtained using Tukey's honestly significant difference post hoc test following one-way analysis of variance. *p<0.05 was considered statistically significant. CEJ, cementoenamel junction; M1, maxillary first molar.

Parameter	Height	Class I vs Class II		Class I vs Class III		Class II vs Class III	
		t statistics	p-value	t statistics	p-value	t statistics	p-value
Total thickness	11 mm apical to M1 CEJ	2.85	0.052	2.98	0.041*	4.32	0.005*
	13 mm apical to M1 CEJ	2.71	0.062	3.05	0.037*	4.18	0.007*
Cortical thickness	11 mm apical to M1 CEJ	2.10	0.145	1.52	0.318	2.68	0.084
	13 mm apical to M1 CEJ	1.95	0.178	1.38	0.372	2.44	0.112
Available bone thickness	11 mm apical to M1 CEJ	2.63	0.071	2.79	0.055	4.05	0.009*
	13 mm apical to M1 CEJ	2.58	0.079	2.71	0.062	3.92	0.011*

## Discussion

The present retrospective CBCT study evaluated the IZC bone thickness at standardized levels of 11 mm and 13 mm apical to the cementoenamel junction of the maxillary first molar to determine its suitability for extra-alveolar TAD placement. Three-dimensional assessment using CBCT provides a more accurate representation of the IZC anatomy than conventional two-dimensional radiographs and facilitates individualized treatment planning by minimizing the risk of root injury and maxillary sinus perforation [4,6]. The findings of the present study demonstrated that both total bone thickness and available bone thickness increased at the 13 mm level compared with the 11 mm level, indicating that the more apical region of the IZC offers a more favorable site for TAD placement.

An important finding of the present investigation was the significant difference in IZC bone thickness between the male and female subjects. Males exhibited significantly greater total, cortical, and available bone thicknesses than females at both measurement levels. These findings are consistent with those reported by Murugesan and Sivakumar [3], who observed greater IZC dimensions in males, which they attributed to generally larger craniofacial skeletal dimensions and thicker cortical bones. Similar observations have also been reported by Gibas-Stanek et al. [8] and Liou et al. [9], who demonstrated that males possess significantly greater cortical and total bone thickness in the posterior maxilla, contributing to the improved primary stability of extra-alveolar skeletal anchorage devices. The greater bone dimensions observed in males may therefore provide a wider safety margin during TAD insertion; however, individualized radiographic assessment remains essential regardless of sex. The present findings are in agreement with a recent systematic review and meta-regression by Hijazi Muwaquet et al. [10], who analyzed 17 CBCT studies and reported that insertion height and angulation significantly influence IZC bone thickness. The authors recommended the upper first and second molar region with an insertion height of approximately 9.9 mm and an 80^o^ angulation as the optimal site for miniscrew placement, emphasizing the importance of CBCT-based individualized assessment for achieving greater primary stability and reducing complications.

The present study also demonstrated significant differences in the total and available bone thickness among the skeletal malocclusion groups. Skeletal Class III subjects exhibited the greatest bone thickness, followed by Class I and Class II subjects, whereas the cortical bone thickness did not differ significantly among the groups. These findings are in close agreement with those of Lee et al. [11], who reported significantly greater IZC dimensions in Class III growing individuals in the apical direction. This observation is biologically plausible because Class III patients generally exhibit a broader maxillary buttress and a more robust zygomatic process morphology than Class II individuals. The lack of significant differences in cortical bone thickness suggests that the skeletal pattern predominantly influences the quantity of cancellous bone rather than cortical bone dimensions. From a clinical perspective, patients with skeletal Class II malocclusion may require more careful selection of the insertion site and screw length because of the relatively reduced available bone. This observed structural robustness in Class III cases aligns with broader tomographic evidence demonstrating distinct jaw volumetric variations across different sagittal skeletal patterns, as reported by Alhawasli et al. [12].

Tavares et al. [13] reported that bone availability was lower in the apical region, particularly in Class II subjects, and recommended the use of shorter miniscrews and a reduced insertion angle when bone availability is limited. In contrast, an earlier CBCT study by Tavares et al. [14] found no significant influence of sagittal or vertical skeletal patterns on the IZC thickness. The discrepancy between these findings may be attributed to differences in the measurement protocols, insertion heights and angulations, imaging techniques, and population characteristics. Nevertheless, both studies emphasized the importance of individualized CBCT evaluation before extra-alveolar miniscrew placement to ensure adequate bone support and to avoid maxillary sinus perforation.

The measurements obtained at 13 mm apical to the cementoenamel junction consistently demonstrated greater total and available bone thicknesses than those recorded at 11 mm. This finding supports previous CBCT investigations by Sharan et al. [6], Song et al. [15], Hijazi Muwaquet et al. [10], and Al Amri et al. [16], who reported an increase in bone availability in the superior aspect of IZC. The increased bone thickness at this level may be attributed to the anatomical transition toward the zygomatic buttress, where the cortical and cancellous bones become progressively thicker. Clinically, this suggests that insertion of extra-alveolar TADs at approximately 13 mm from the cementoenamel junction while maintaining the recommended insertion angulation may improve primary stability. Nevertheless, because the proximity of the maxillary sinus also increases, preoperative CBCT evaluation remains indispensable for safe placement.

The greater total and available bone thickness observed in the present study, particularly at 13 mm apical to the cementoenamel junction, may contribute to the improved primary stability of the infrazygomatic miniscrews. Although the present study did not evaluate the clinical outcomes, Uribe et al. [17] reported an overall failure rate of 21.8% for infrazygomatic miniscrews and emphasized that careful selection of the insertion site and adequate bone support are essential for achieving predictable skeletal anchorage. Therefore, individualized CBCT assessment remains indispensable for optimizing miniscrew placement and minimizing the risk of failure. The results of the present study reinforce the recommendation that CBCT be considered during treatment planning for IZC miniscrews. Consistent with the systematic review by Stasiak and Adamska [18], individualized three-dimensional assessment allows clinicians to identify the optimal insertion site and angulation based on patient-specific anatomy, thereby improving the safety and predictability of extra-alveolar skeletal anchorage.

Strengths

The present study has several methodological strengths. A relatively large sample size, inclusion of both sexes and all skeletal malocclusion groups, standardized measurement levels, blinded assessment by independent examiners, and rigorous statistical analysis improved the internal validity of the study. Furthermore, CBCT enables accurate assessment of three-dimensional bone morphology, overcoming the inherent limitations of conventional radiography. These findings contribute valuable population-specific anatomical data that may assist orthodontists in selecting the optimal insertion sites for extra-alveolar TADs in routine clinical practice.

Limitations

However, these findings should be interpreted in the light of certain limitations. Because this was a retrospective, single-center study, the results may not be generalizable to all ethnic populations. The facial growth pattern, body mass index, age-related bone remodeling, and bone density were not evaluated and may influence the IZC morphology. In addition, the study assessed anatomical bone availability without evaluating the clinical success or long-term stability of the TADs. Future prospective multicenter studies incorporating bone density assessment, finite element analysis, and longitudinal clinical outcomes would provide a more comprehensive understanding of the factors influencing the success of IZC anchorage. Moreover, the thickness of the IZC was not evaluated for the different vertical patterns.

Clinical implications

The findings of this study suggest that the IZC provides adequate bone for extra-alveolar TAD placement, particularly at 13 mm apical to the cementoenamel junction, where greater total and available bone thickness was observed. The demonstrated variations according to sex and skeletal malocclusion highlight the importance of individualized CBCT evaluations before TAD placement. These data may assist clinicians in selecting the optimal insertion site, angulation, and screw length, thereby improving the primary stability and minimizing the risk of root damage and maxillary sinus perforation.

## Conclusions

Within the limitations of this retrospective CBCT study, the IZC was found to provide adequate bone for extra-alveolar TAD placement, with greater total and available bone thickness observed at 13 mm than at 11 mm apical to the cementoenamel junction. Male subjects and skeletal Class III individuals exhibited significantly greater bone thickness than females and Class II subjects. These findings support the use of individualized CBCT assessments for optimizing IZC TAD placement and improving the safety and predictability of orthodontic skeletal anchorage.
